# Microtubule-associated protein 1 A and tubby act independently in regulating the localization of stereocilin to the tips of inner ear hair cell stereocilia

**DOI:** 10.1186/s13041-022-00966-z

**Published:** 2022-09-14

**Authors:** Song Yi Youn, Hyehyun Min, Se Rok Jeong, Jiahn Lee, Seok Jun Moon, Jinwoong Bok, Chul Hoon Kim

**Affiliations:** 1 Department of Pharmacology, BK21 PLUS Project for Medical Science, Brain Research Institute, Yonsei University College of Medicine, 03722 Seoul, Korea; 2 Department of Anatomy, BK21 PLUS Project for Medical Science, Yonsei University College of Medicine, 03722 Seoul, Korea; 3 Department of Oral Biology, BK21 FOUR Project, Yonsei University College of Dentistry, 03722 Seoul, Korea

**Keywords:** Tubby, MAP1A, Stereocilia, Stereocilin, Cochlear

## Abstract

*Tubby* mice exhibit hearing impairment due to the loss of stereocilin from the tip regions that connect the tallest stereocilia of the outer hair cells (OHCs) to the tectorial membrane. Stereocilin is an essential stereociliary protein in the OHCs, the mutation of which in humans causes autosomal recessive non-syndromic deafness. *Map1a* is a modifier of tubby hearing (*moth1*), and its wild-type allele, rather than the *moth1* allele from the C57BL/6 J strain, restores stereocilin localization to the stereocilia and rescues the hearing impairment of *tubby* mice. The mechanism by which MAP1A accomplishes this is unclear, partly due to ambiguity regarding whether the *tubby* mutation is a true null. We therefore generated *Tub*-null (*Tub*^−/−^) mice by deleting exon 3 and found that they exhibit hearing impairment like that of *tubby* mice, suggesting the *tubby* mutation is a loss-of-function mutation with regard to hearing. When we crossed *Tub*^−/−^ mice with AKR mice that have wild-type *Map1a* alleles, we found that wild-type MAP1A restores stereocilin localization to the tips of stereocilia and rescues hearing impairment. These data suggest MAP1A does not require interaction with tubby protein in maintaining stereocilin at the tips of stereocilia and that OHCs use two independent molecules—MAP1A and tubby—to doubly ensure proper stereocilin localization.

**Supplementary Information:**

The online version contains supplementary material available at 10.1186/s13041-022-00966-z.

*Tubby* mice show obesity, blindness and deafness [1]. The *tubby* mutation is a G-to-T transversion that causes a splicing defect in the 3’-end of the *Tub* gene [2], but the molecular mechanisms underlying these phenotypes in *tubby* mice have been remained mysterious for a long time. We recently revealed the molecular mechanism by which *tubby* mice develop hearing impairment. Stereocilin, which should be localized to the tips of auditory stereocilia, is mislocalized in *tubby* mice [3]. Stereocilin is essential for maintaining the physical links between the outer hair cell (OHC) stereocilia and the tectorial membrane (TM), which is called TM-attachment links (TMALs). *Strc* knockout (KO) mice have defective TMALs, which are essential for mechanotransduction, leading to hearing impairment [4]. Naturally, *tubby* mice phenocopy *Strc* KO mice [3]. *Map1a* is a modifier of tubby hearing (*moth1*) [5] and, intriguingly, its wild-type allele, rather than the *moth1* allele from C57BL/6 J mice, rescues the hearing impairment of *tubby* mice [3, 5]. Because tubby protein is highly expressed in neurons including spiral ganglion neurons that innervate the hair cells of the Organ of Corti, the mechanism of hearing rescue by microtubule-associated protein 1 A (MAP1A) was considered neuronal. For example, some expected it was related to MAP1A’s ability to bind the PSD95 postsynaptic protein [6, 7]. The recent progress showed that MAP1A possesses distinct and unexpected roles in the inner ear hair cell system; MAP1A regulates the stereociliary localization of stereocilin [3]. Due to the microscopic size and inaccessibility of the inner ear stereocilia, we have little information about the molecular interactions that support the localization of the stereociliary proteins (e.g., stereocilin, CDH23, PCDH15, TMC, etc.) required for normal hearing. These points suggest further study of MAP1A’s role will help clarify how the localization of stereociliary proteins is regulated.

First, we wanted to address whether MAP1A acts independently of mutant tubby protein to show that MAP1A itself regulates stereocilin localization. While we were unable to detect any tubby protein in the OHCs of *tubby* mice [3], the presence of *Tub* transcript and tubby protein were reported in the retina and the cochlear of *tubby* mice [5, 8]. To solve this problem, we generated *Tub* conditional KO mice in which exon 3 of the *Tub* gene is flanked by loxP sites. The resulting *Tub*^*flox/flox*^ mice were crossed with a global “Cre-deleter” mice (*E2a-Cre*) to produce homozygous null offspring (Fig. 1 A, B). In a previous study, targeted deletion of the *Tub* gene was found to causes retinal degeneration and obesity, suggesting the *tubby* mutation is a loss-of-function mutation at least in terms of these two *tubby* mouse phenotypes [9]. But, no one has reported whether *Tub*-null (*Tub*^−/−^) mice also show hearing impairment. Immunofluorescence staining in *Tub*^−/−^ mice we generated revealed that stereocilin proteins in the OHC stereocilia disappeared (Fig. 1C). Accordingly, we found that *Tub*^−/−^ mice show elevated auditory brainstem response (ABR) thresholds like the ABR shifts observed in *tubby* mice [3] (Fig. 1D). *Tub*^−/−^ mice also showed elevated thresholds and reduced amplitudes of their distortion product otoacoustic emissions (DPOAEs), which are used to assess OHC integrity (Fig. 1E) (see Additional file 1 for the detailed methods and Additional file 2 for amplitude of DPOAEs). These findings confirm hearing impairment in *Tub*^−/−^ mice and suggest that the hearing impairment of *tubby* mice is due to a loss-of-function of *Tub* gene. We next examined whether the deletion of *Tub* gene in the inner ear causes hearing impairment using *Tub*^*flox/flox*^; *Pax2*-*Cre* mice. We observed that deletion of *Tub* in the inner ear is responsible for hearing impairment in *tubby* mice (Fig. 1F, G; Additional file 2 for amplitude of DPOAEs and immunofluorescence staining of stereocilin), confirming that the site of action of tubby is cochlear. Ultimately, we explored whether wild-type MAP1A can rescue both hearing impairment and stereocilin mislocalization in *Tub*-deficient mice. The genetic background of *Tub*^−/−^ mice is C57BL/6 J that has a nonprotective variant of *Map1a* (*Tub*^−/−^; *Map1a*^*B6*^). Sequential mating *Tub*^−/−^ mice with AKR mice produced offspring that lack *Tub* but have a wild-type, protective *Map1a* allele (*Tub*^−/−^; *Map1a*^*AKR*^). We found that *Tub*^−/−^; *Map1a*^*AKR*^ mice show the recovery of ABR threshold shifts and loss of DPOAE amplitudes (Fig. 1D, E) similar to that reported in *tub/tub*; *Map1a*^*AKR*^ mice [3] and restore the localization of stereocilin to the tips of the tallest OHC stereocilia (Fig. 1H). These findings indicate that MAP1A by itself may regulate the localization of stereocilin. Because we did not look for changes in the level of stereocilin protein in the OHCs, we cannot rule out the possibility that TUB or MAP1A might affect stereocilin expression or stability. This would obviously also affect its localization to the stereocilia. It also remains unclear whether the MAP1A-mediated localization of stereocilin to the tips of the stereocilia in *Tub*^*−/−*^;*Map1a*^*AKR*^ mice shows an age-dependent decline and it is more vulnerable to aging or environmental stressors such as noise.

MAP1A plays important roles in stabilizing microtubules in neurons [10]. However, its roles in other cell types are obscure. The *Map1a* allele from AKR mice also reduces photoreceptor degeneration in *Tulp1*- and *Tub*-deficient mice [11]. However, we observed that *Tub*^−/−^; *Map1a*^*AKR*^ mice still show obesity (unpublished data), suggesting that sensory cells may share pathologic mechanisms related to MAP1A. The detailed roles of MAP1A in the cell surface specializations of photoreceptors and cochlear hair cells are waiting to be discovered. Because there has been no report showing the presence of microtubule in the stereocilia, new function of MAP1A other than the conventional role of stabilizing microtubule can be expected. It is unclear why two seemingly unrelated proteins, MAP1A and tubby, contribute to the proper localization of a single stereociliary molecule. Stereocilia are nano-scale structures. However, they have fine cytoskeletal structures and complex protein interaction networks like primary cilia or neuronal postsynaptic densities. Stereocilin might be a core member of the stereociliary protein interactome whose localization is so important to normal hearing that two mechanisms are necessary. In addition, the independent contributions of MAP1A and tubby to the localization of stereocilin suggest it may be important to consider a “two-hit” mechanism when assessing the roles of stereociliary proteins in hearing impairment.

**Fig. 1:**
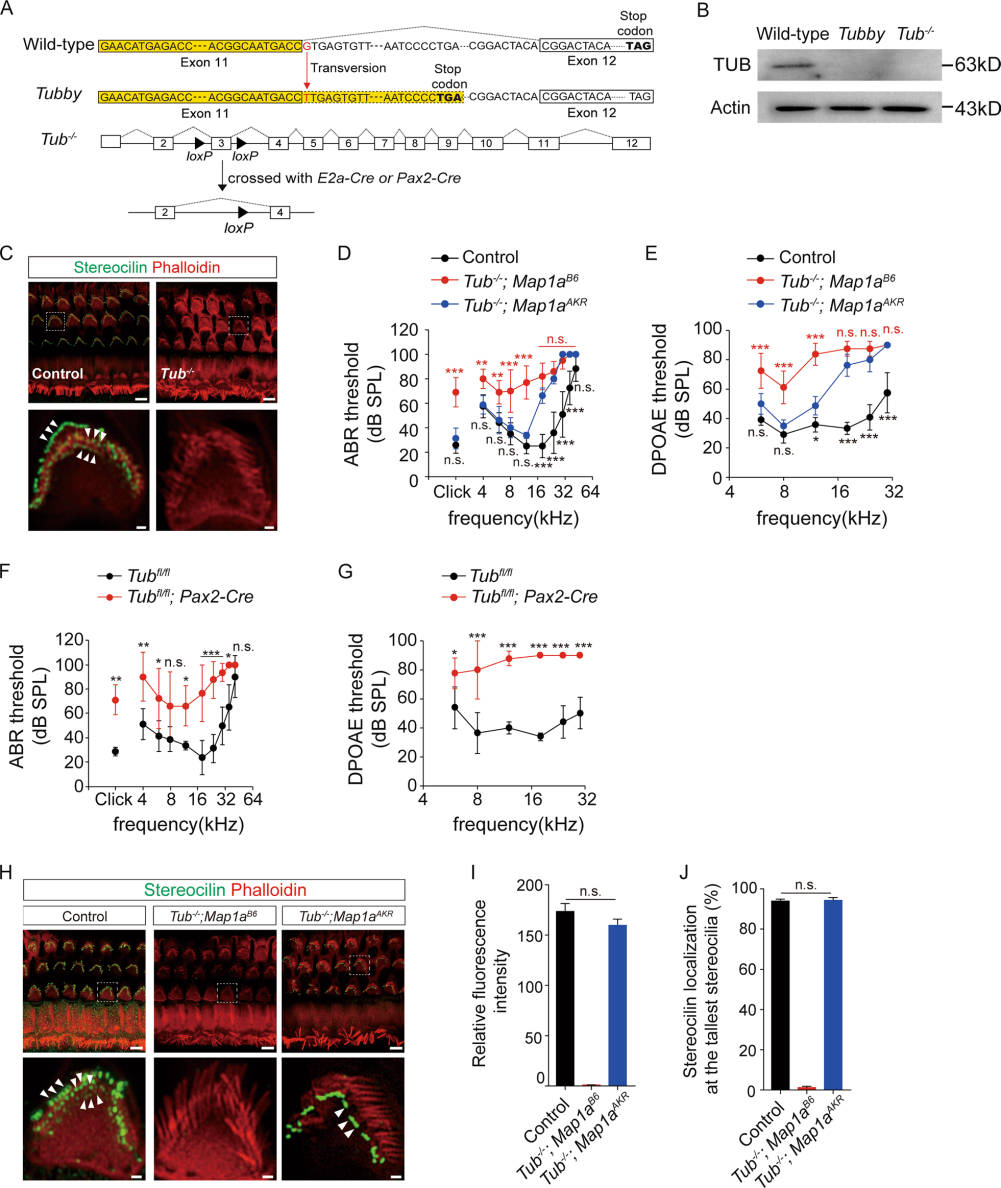
Wild-type MAP1A can rescue hearing impairment and preserve the localization of stereocilin to the tips of stereocilia in the absence of tubby protein. **A** *Tub*^*flox/flox*^ mice were crossed with *E2a-Cre* mice expressing *Cre* in germ cells to produce null mutant mice. **B** Absence of a tubby protein band at the expected molecular size of approximately 63 kDa in western blots of the brain lysates from *tubby* mice and *Tub*-null mice. **C** Immunostaining of stereocilin in the stereocilia of 5–7-week-old control B6J (wild-type or *Tub*^±^) and *Tub*-null mice. A representative image from one of three experiments is shown. Arrows indicate the localization of stereocilin in the stereocilia. Scale bars: low-magnification images, 5 mm; high-magnification images, 0.5 mm. **D**, **E** ABR (D) and DPOAE (E) were measured in 5–7-week-old control (wild-type or *Tub*^±^), *Tub*^−/−^ (*Tub*^−/−^; *Map1a*^*B6*^) and *Tub*^−/−^; *Map1a*^*AKR*^ mice. *Tub*-null mice were crossed with AKR/N mice which have a *Map1a*^*AKR*^ allele. *Tub*^±^; *Map1a*^*AKR*^ mice were crossed together to generate *Tub*^−/−^; *Map1a*^*AKR*^ mice. *P < 0.05, **P < 0.01, ***P < 0.001 compared to *Tub*^−/−^; *Map1a*^*AKR*^ mice. *n* = 4–6. **F**, **G** *Tub*^*flox/flox*^ mice were crossed with *Pax2*-*Cre* mice. *Tub*^*+/flox*^; *Pax2*-*Cre* mice were crossed together to generate *Tub*^*flox/flox*^; *Pax2*-*Cre* mice. ABR (F) and DPOAE (G) were measured in 5–7-week-old *Tub*^*flox/flox*^ and *Tub*^*flox/flox*^; *Pax2*-*Cre* mice. *P < 0.05, **P < 0.01, ***P < 0.001 compared to *Tub*^*flox/flox*^ mice. *n* = 4. **H** Immunostaining of stereocilin was performed in control B6J (wild-type or *Tub*^±^), *Tub*^−/−^ (*Tub*^−/−^; *Map1a*^*B6*^) and *Tub*^−/−^; *Map1a*^*AKR*^ mice. A representative image from one of three experiments is shown. Arrows indicate the localization of stereocilin in the stereocilia. **I** Quantification of stereocilin fluorescence intensity in the tallest row of stereocilia. Average fluorescent intensity was measured in 13–20 hair cells per mouse and averaged across three mice for each group. Images were analyzed using ImageJ. **J** Quantification of the number of the tallest stereocilia with stereocilin at their tips. Eight to ten hair cells in each mouse were counted and averaged across three mice for each group. All data are presented as means ± SEM

## Supplementary Information

**Additional file 1:** Materials and methods.

**Additional file 2:** **Figure S1.** Measurements of DPOAE amplitudes. **Figure S2.** Disappearance of stereocilin from hair cell stereocilia in Tubflox/flox; Pax2-Cre mice.

## Data Availability

All data and materials are available upon requests.

## Abbreviations

MAP1A: Microtubule associated protein 1 A
OHC: Outer hair cell
TM: Tectorial membrane
