# Supplementary material for: Microtubule-associated protein 1 A and tubby act independently in regulating the localization of stereocilin to the tips of inner ear hair cell stereocilia
Source: Mol Brain. 2022 Sep 14;15:80. doi: 10.1186/s13041-022-00966-z (PMC9472429; doi:10.1186/s13041-022-00966-z)
Supplement: Supplementary file 1 — Additional file 1: Materials and methods. [file 13041_2022_966_MOESM1_ESM.docx]

Additional file 1.

**Materials and Methods**

**Mice**

*Tub*-floxed mice (B6J) were generated using CRISPR technology by inserting two loxP cassettes on either side of exon 3 of the *Tub* gene (Applied StemCell). Wild-type inbred AKR/N mice expressing *Map1a^AKR^* were purchased from Japan SLC, Inc (Cat. # SLC-M-0248; Shizuoka, Japan). *E2a-Cre* mice (B6.FVB-Tg(EIIa-cre)C5379Lmgd/J) were purchased from Jackson Laboratory and backcrossed with B6J mice more than 4 times. *Tub*-floxed mice were crossed with *E2a-Cre* mice expressing Cre in germ cells to produce *Tub*-null mice. *Tub*-floxed mice were crossed with *Pax2-Cre* (B6J) mice to delete *Tub* in the inner ear. In the first cross, *Tub*-null mice were bred with AKR/N mice to generate *Tub*^+/-^; *Map1a^AKR^* mice. Wild-type, *Tub*^+/-^, and *Tub*^-/-^ denote mice with two *MAP1a^B6^* alleles, while *Tub^-/-^*;*Map1a^AKR^* denotes mice with either one *MAP1a^B6^* and one *MAP1a^AKR^* allele or two alleles of *MAP1a^AKR^*. In the second cross, *Tub*^+/-^; *Map1a^AKR^* mice were crossed together to generate the desired *Tub*^-/-^; *Map1a^AKR^* mice. *Tub^-/-^*;*Map1a^AKR^* mice were genotyped and the mice with one *Map1a^AKR^* allele were used in this experiment. The mice were maintained in a temperature- and humidity-controlled, specific pathogen-free (SPF) environment with a 12 h/12 h light/dark cycle control. All animal protocols were approved (No. 2020-0226) by the Institutional Animal Care and Use Committee at Yonsei University College of Medicine with NIH guidelines.

**Antibodies**

Anti-stereocilin antibodies raised earlier in the laboratory were used [1]. Briefly, a stereocilin antibody was generated by injecting rabbits with a synthetic peptide CFLSPEELQSLVPLSD, corresponding to amino acids 970–985 of the mouse stereocilin protein.

**Immunofluorescence staining**

Immunofluorescence staining of the whole-mount cochlea was performed as previously described [1]. The mouse inner ears were quickly dissected and immediately fixed with 4% paraformaldehyde in PBS for 30 min at room temperature (RT) with gentle agitation. The cochlear epithelium was micro-dissected and further fixed in 4% paraformaldehyde in PBS for 30 min at RT. The sample was then blocked with 20% goat serum in PBS for 1 h at RT and permeabilized with 0.5% Triton X-100 in PBS for 30 min. After overnight incubation with primary antibodies diluted in 1% BSA in PBS at 4 °C, secondary antibodies (Alexa Fluor 488- or 568-conjugated secondary antibodies, Thermo Fisher Scientific, IL, USA) and phalloidin (Thermo Fisher Scientific, IL, USA) were applied for 1 h at RT. These samples were then washed, mounted on a slide glass with ProLong Gold anti-fade reagent (Thermo Fisher Scientific, IL, USA). Imaging was performed using a LSM880 with an airy-scan detector (Carl Zeiss, Jena, Germany).

**Auditory Brainstem Response (ABR)**

ABR measurements were performed in a sound-proof chamber using Tucker-Davis Technologies (TDT) RZ6 digital signal processing hardware and the BioSigRZ software package (Alachua, FL, USA). Under anesthesia, sub-dermal electrodes were positioned at the vertex and ventrolateral to the right and left ear of mice. Calibrated click stimulus (10 µs duration) or tone burst stimuli (5 ms duration) at 4, 6, 8, 12, 18, 24, 30, 36, 42 kHz were produced using the SigGenRZ software package with an RZ6 digital signal processor and delivered to the ear canal through a multi-field 1 (MF1) magnetic speaker (TDT). The stimulus intensity was increased from 10 to 95 dB SPL in 5 dB steps. The recorded signals were filtered using a 0.3–3 kHz band-pass filter and the ABR waveforms in response to 512 tone bursts were averaged. The ABR thresholds for each frequency were determined using the BioSigRZ software.

**Distortion Product Otoacoustic Emissions (DPOAE)**

DPOAEs measurements were performed using a combination TDT microphone-speaker system. The primary stimulus tones were produced using an RZ6 digital signal processor and the SigGenRZ software and delivered through a custom probe containing an ER 10B+ microphone (Etymotic, Elk Grove Village, IL, USA) and MF1 speakers positioned in the ear canal. For DPOAE amplitude, the primary tones were set at a frequency ratio (f2/f1) of 1.2 with target frequencies at 6, 12, 16, 18, 22, and 24 kHz. The f1 intensity level (L1) was 65 dB SPL and the f2 intensity level (L2) was 55 dB SPL which is 10 dB SPL below the L1. For the DPOAE thresholds, the frequency ratio (f2/f1) of the main tones was set at 1.2 with target frequencies of 6, 8, 12, 18, 24, and 30 kHz. The f1 and f2 intensities were adjusted to identical levels between 20 to 80 dB SPL. The resultant sounds in response to the primary tones were received through the ER 10B+ microphone and recorded using the RZ6 digital signal processor. At each primary tone for the DP grams, the Fast Fourier Transform (FFT) was performed using the BioSigRZ software package to determine the average spectra of the two primaries, the 2f1-f2 distortion products, and the noise floors at each primary tone.

**Statistical Analysis**

All results are expressed as means ± standard error of the mean. Statistical comparisons were made with the two-way RM analysis of variance (ANOVA) with Bonferroni corrections for multiple comparisons for the ABRs and DPOAEs using Prism 8.0 (GraphPad, San Diego, CA, USA). Statistical significance is indicated in the figures as n.s., non-significant (P > 0.05), **P* < 0.05, ***P* < 0.01, ****P* < 0.001.

1. Han W, Shin JO, Ma JH, Min H, Jung J, Lee J, et al. Distinct roles of stereociliary links in the nonlinear sound processing and noise resistance of cochlear outer hair cells. Proc Natl Acad Sci U S A. 2020;117(20):11109-17.
