# Supplementary material for: Microtubule-associated protein 1 A and tubby act independently in regulating the localization of stereocilin to the tips of inner ear hair cell stereocilia
Source: Mol Brain. 2022 Sep 14;15:80. doi: 10.1186/s13041-022-00966-z (PMC9472429; doi:10.1186/s13041-022-00966-z)
Supplement: Supplementary file 2 — Additional file 2: Figure S1. Measurements of DPOAE amplitudes. Figure S2. Disappearance of stereocilin from hair cell stereocilia in Tubflox/flox; Pax2-Cre mice. [file 13041_2022_966_MOESM2_ESM.docx]

Additional file 2.


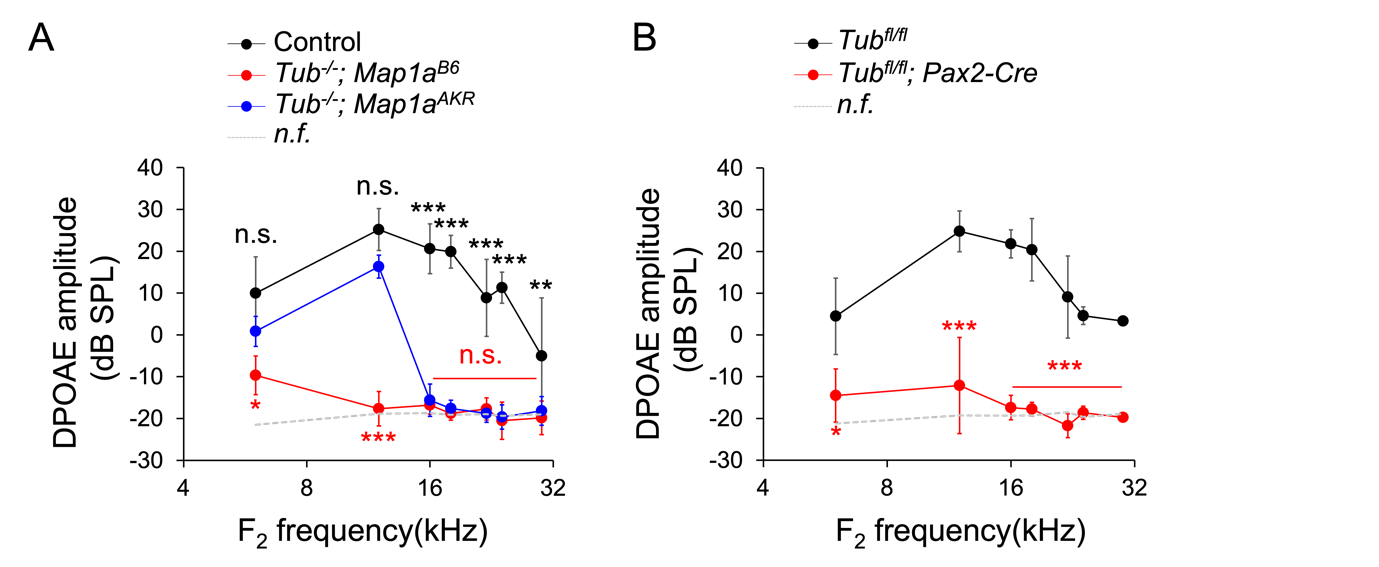


C

**Figure S1.** **Measurements of DPOAE amplitudes**. The amplitudes of DPOAEs were measured in 5-7-week-old mice. Values and error bars reflect means ± SEM. **P* < 0.05, ***P* < 0.01, ****P* < 0.001 compared to *Tub*^-/-^; *Map1a^AKR^* mice (left) and **P* < 0.05, ****P* < 0.001 compared to *Tub^flox/flox^* (right). *n.f.* : noise floor

**
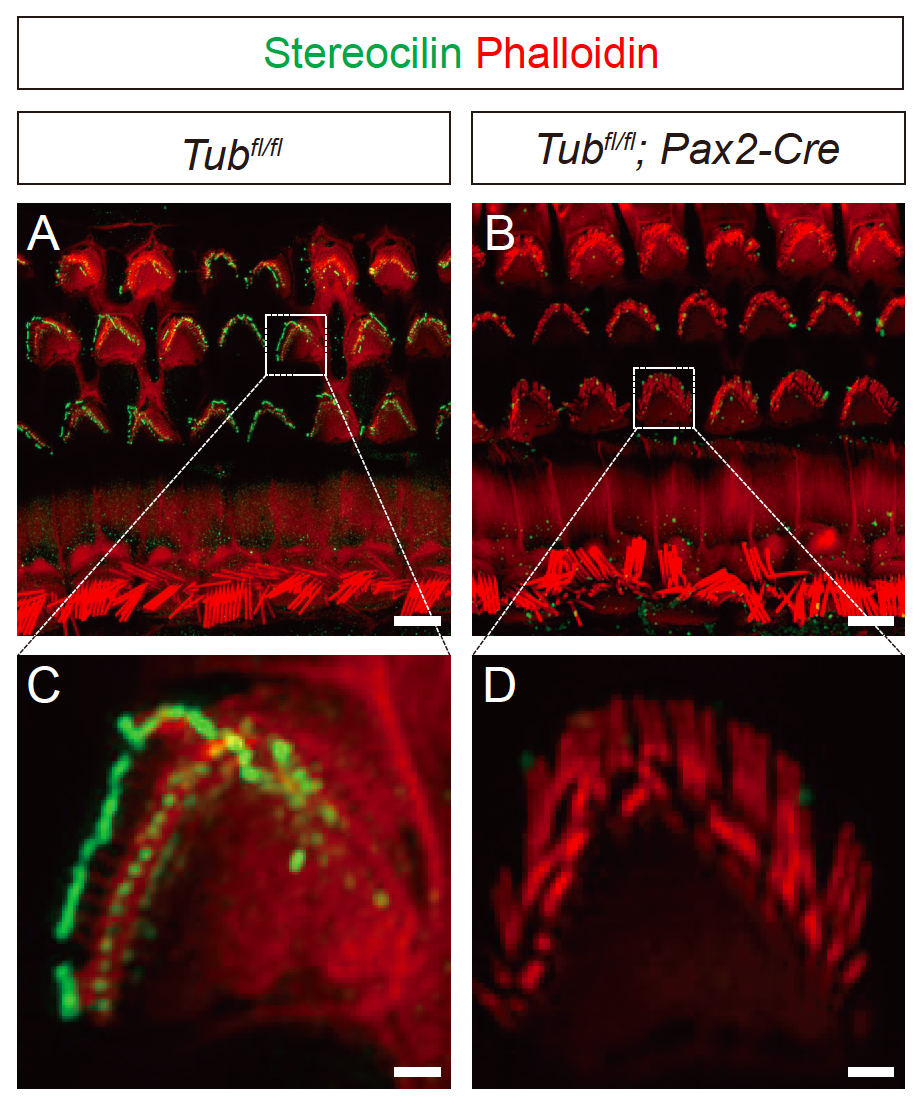
**

**Figure S2. Disappearance of stereocilin from hair cell stereocilia in *Tub^flox/flox^*; *Pax2-Cre* mice. A, B** Immunostaining of stereocilin was performed in control B6J (wild-type or *Tub*^+/-^) and *Tub^flox/flox^*; *Pax2-Cre* mice. A representative image from one of three experiments is shown. **C, D** Magnified view of the hair bundles of hair cells. Arrows indicate the localization of stereocilin in the stereocilia. Scale bars: A and B, 5 μm; C and D, 0.5 μm.
